# Sex-specific relevance of diabetes to occlusive vascular and other mortality: a collaborative meta-analysis of individual data from 980 793 adults from 68 prospective studies

**DOI:** 10.1016/S2213-8587(18)30079-2

**Published:** 2018-07

**Authors:** Prospective Studies Collaboration and Asia Pacific Cohort Studies Collaboration, L Gnatiuc, WG Herrington, J Halsey, J Tuomilehto, X Fang, HC Kim, D De Bacquer, AJ Dobson, MH Criqui, DR Jacobs, DA Leon, SAE Peters, H Ueshima, P Sherliker, R Peto, R Collins, RR Huxley, JR Emberson, M Woodward, S Lewington, N Aoki, H Arima, E Arnesen, A Aromaa, G Assmann, DL Bachman, C Baigent, H Bartholomew, A Benetos, C Bengtsson, D Bennett, C Björkelund, H Blackburn, K Bonaa, E Boyle, R Broadhurst, J Carstensen, L Chambless, Z Chen, SK Chew, R Clarke, C Cox, JD Curb, R D'Agostino, C Date, G Davey Smith, G De Backer, SS Dhaliwal, XF Duan, P Ducimetiere, S Duffy, H Eliassen, P Elwood, J Empana, MH Garcia-Palmieri, P Gazes, GG Giles, C Gillis, U Goldbourt, DF Gu, M Guasch-Ferre, L Guize, L Haheim, C Hart, S Hashimoto, T Hashimoto, D Heng, I Hjermann, SC Ho, M Hobbs, D Hole, I Holme, H Horibe, A Hozawa, F Hu, K Hughes, M Iida, K Imai, Y Imai, H Iso, R Jackson, K Jamrozik, SH Jee, G Jensen, CQ Jiang, NB Johansen, T Jorgensen, P Jousilahti, M Kagaya, J Keil, J Keller, IS Kim, Y Kita, A Kitamura, Y Kiyohara, P Knekt, M Knuiman, M Kornitzer, D Kromhout, R Kronmal, TH Lam, M Law, J Lee, P Leren, D Levy, YH Li, L Lissner, R Luepker, M Luszcz, S MacMahon, H Maegawa, M Marmot, Y Matsutani, T Meade, J Morris, R Morris, T Murayama, Y Naito, K Nakachi, M Nakamura, T Nakayama, J Neaton, PJ Nietert, Y Nishimoto, R Norton, A Nozaki, T Ohkubo, A Okayama, WH Pan, P Puska, N Qizilbash, A Reunanen, E Rimm, A Rodgers, S Saitoh, K Sakata, S Sato, P Schnohr, H Schulte, R Selmer, D Sharp, X Shifu, K Shimamoto, M Shipley, H Silbershatz, p Sorlie, P Sritara, I Suh, SE Sutherland, P Sweetnam, A Tamakoshi, H Tanaka, T Thomsen, S Tominaga, M Tomita, S Törnberg, H Tunstall-Pedoe, A Tverdal, H Ueshima, E Vartiainen, N Wald, SG Wannamethee, TA Welborn, P Whincup, G Whitlock, W Willett, J Woo, ZL Wu, SX Yao, J Yarnell, T Yokoyama, N Yoshiike, XH Zhang

## Abstract

**Background:**

Several studies have shown that diabetes confers a higher relative risk of vascular mortality among women than among men, but whether this increased relative risk in women exists across age groups and within defined levels of other risk factors is uncertain. We aimed to determine whether differences in established risk factors, such as blood pressure, BMI, smoking, and cholesterol, explain the higher relative risks of vascular mortality among women than among men.

**Methods:**

In our meta-analysis, we obtained individual participant-level data from studies included in the Prospective Studies Collaboration and the Asia Pacific Cohort Studies Collaboration that had obtained baseline information on age, sex, diabetes, total cholesterol, blood pressure, tobacco use, height, and weight. Data on causes of death were obtained from medical death certificates. We used Cox regression models to assess the relevance of diabetes (any type) to occlusive vascular mortality (ischaemic heart disease, ischaemic stroke, or other atherosclerotic deaths) by age, sex, and other major vascular risk factors, and to assess whether the associations of blood pressure, total cholesterol, and body-mass index (BMI) to occlusive vascular mortality are modified by diabetes.

**Results:**

Individual participant-level data were analysed from 980 793 adults. During 9·8 million person-years of follow-up, among participants aged between 35 and 89 years, 19 686 (25·6%) of 76 965 deaths were attributed to occlusive vascular disease. After controlling for major vascular risk factors, diabetes roughly doubled occlusive vascular mortality risk among men (death rate ratio [RR] 2·10, 95% CI 1·97–2·24) and tripled risk among women (3·00, 2·71–3·33; χ^2^ test for heterogeneity p<0·0001). For both sexes combined, the occlusive vascular death RRs were higher in younger individuals (aged 35–59 years: 2·60, 2·30–2·94) than in older individuals (aged 70–89 years: 2·01, 1·85–2·19; p=0·0001 for trend across age groups), and, across age groups, the death RRs were higher among women than among men. Therefore, women aged 35–59 years had the highest death RR across all age and sex groups (5·55, 4·15–7·44). However, since underlying confounder-adjusted occlusive vascular mortality rates at any age were higher in men than in women, the adjusted absolute excess occlusive vascular mortality associated with diabetes was similar for men and women. At ages 35–59 years, the excess absolute risk was 0·05% (95% CI 0·03–0·07) per year in women compared with 0·08% (0·05–0·10) per year in men; the corresponding excess at ages 70–89 years was 1·08% (0·84–1·32) per year in women and 0·91% (0·77–1·05) per year in men. Total cholesterol, blood pressure, and BMI each showed continuous log-linear associations with occlusive vascular mortality that were similar among individuals with and without diabetes across both sexes.

**Interpretation:**

Independent of other major vascular risk factors, diabetes substantially increased vascular risk in both men and women. Lifestyle changes to reduce smoking and obesity and use of cost-effective drugs that target major vascular risks (eg, statins and antihypertensive drugs) are important in both men and women with diabetes, but might not reduce the relative excess risk of occlusive vascular disease in women with diabetes, which remains unexplained.

**Funding:**

UK Medical Research Council, British Heart Foundation, Cancer Research UK, European Union BIOMED programme, and National Institute on Aging (US National Institutes of Health).

## Introduction

Worldwide, the age-standardised prevalence of diabetes more than doubled between 1980 and 2014, with the greatest relative and absolute increases seen in low-income and middle-income countries.^1^ Results from a previous large-scale collaborative meta-analysis of prospective studies done mostly in high-income countries showed that diabetes was associated with an approximate doubling in all-cause mortality risk, with the greatest excess due to deaths from occlusive vascular diseases (eg, ischaemic heart disease and ischaemic stroke).^2^, ^3^ In those studies, the proportional increase in occlusive vascular risk associated with diabetes was higher among women than among men.^3^ Similarly, findings from a large Chinese study showed somewhat larger relative risks associated with diabetes for vascular mortality in women than in men.^4^ However, adjustment for other established vascular risk factors (eg, blood pressure and cholesterol) was not always possible in these studies, so the larger relative risks seen in women could be due to differences in the extent of confounding by these factors. By contrast, in a large study of Mexican adults,^5^ diabetes conferred a much larger overall proportional increase in mortality risk than had been seen in these previous studies, and this relative risk was identical in men and women.

**Research in context**

**Evidence before this study**

Several large prospective studies and meta-analyses of such studies—especially the extensive collaborative meta-analysis by the Emerging Risk Factors Collaboration—have shown that individuals with diabetes are predisposed to increased vascular mortality, particularly from occlusive causes, and a higher relative risk exists among women than among men. Despite these studies, reasons for these sex differences are still unclear; however, they might relate to differential confounding by other established major vascular risk factors, such as blood pressure, total cholesterol, body-mass index (BMI), and smoking status.

**Added value of this study**

Using individual participant-level data from nearly 1 million adults from the Prospective Studies Collaboration and Asia Pacific Cohort Studies Collaboration with complete measurement of established major vascular risk factors, we assessed sex-specific differences in the association between diabetes and occlusive vascular mortality across age groups and established major vascular risk factors. This meta-analysis adjusts for these major risk factors when analysing this association. Even after controlling for total cholesterol, blood pressure, BMI, and smoking status, diabetes conferred a doubling in occlusive vascular mortality risk among men aged 35–89 years, but a tripling in risk among similarly aged women. Among women especially, diabetes death rate ratios were higher at younger ages than at older ages, so that at ages 35–59 years, diabetes was associated with a five to six times increased risk of occlusive vascular mortality. Total cholesterol, blood pressure, and BMI each had continuous log-linear associations with occlusive vascular mortality that were similar in strength among those with and those without diabetes irrespective of sex. Because those with diabetes were at a much higher risk than those without diabetes, however, the absolute relevance of these risk factors to vascular mortality risk was much greater among those with diabetes than those without.

**Implications of the available evidence**

Public health strategies aimed at lifestyle changes (particularly with respect to smoking and adiposity) and wider use of cost-effective drug treatments (eg, statin-based regimens and blood-pressure-lowering drugs) to reduce vascular risks are important among both men and women with diabetes. However, our results suggest that the larger death rate ratios associated with diabetes among women, compared with those among men, do not seem to be explained by these established major vascular risk factors. Future research should consider which emerging risk factors account for the relative excess risk of occlusive vascular disease among women with diabetes.

We aimed to assess whether differences in established risk factors explain higher relative risks of vascular mortality among women than among men. We used individual participant data from adults included in the Prospective Studies Collaboration and the Asia Pacific Cohort Studies Collaboration to estimate sex-specific associations between diabetes and risk of occlusive vascular mortality in subgroups divided by age and other vascular risk factors. We also examined whether the relevance of blood pressure, blood cholesterol, and body-mass index (BMI)—factors that might cause or be caused by diabetes—to occlusive vascular mortality risk varied with the presence or absence of diabetes.

## Methods

### Study population and procedures

This meta-analysis comprises individual participant data collected from prospective observational studies included in the Prospective Studies Collaboration and Asia Pacific Cohort Studies Collaboration (appendix).^6^, ^7^ Baseline data collection ranged from 1949 to 1997 and last follow-up from 1985 to 2002. Details of study selection, data collection, and statistical methods used have been described previously.^6^, ^7^ Participants were included in our analyses if they had mortality follow-up data in the age range 35–89 years and had data on diabetes status, sex, tobacco use (self-reported), blood pressure, total cholesterol, height, and weight (measured in 66 cohorts but self-reported in two studies^8^, ^9^ of US health professionals; appendix).

Diabetes was defined if any of the following criteria were met: self-reported doctor diagnosis, measured fasting plasma glucose concentration of 7 mmol/L or higher (or ≥11·1 mmol/L if postprandial), or fasting serum glucose concentration of 6·1 mmol/L or higher (or ≥10 mmol/L if postprandial). We were not able to differentiate between diabetes types. BMI was calculated as the weight in kg divided by the square of the height in m (kg/m^2^). HDL cholesterol concentration was available in a subset of participants. Analyses were restricted to participants with no self-reported history of ischaemic heart disease or stroke, and with complete information on baseline covariates.

### Outcomes

Causes of death were determined from the reported underlying cause of death from medical death certificates, which is ascribed by use of an international convention.^10^ In most studies, the underlying cause of death was confirmed by a physician using medical records or autopsy findings. We mapped causes of death to the International Statistical Classification of Diseases and Related Health Problems, ninth revision (ICD-9) and categorised them as either vascular (codes 390–459, and 798)—from which occlusive vascular deaths were defined as death from ischaemic heart disease (410–414), ischaemic stroke (433–434), and other atherosclerotic deaths (440, 443, 445), and non-occlusive vascular deaths constituted all remaining vascular codes—or non-vascular deaths, which were separated into cancer deaths (140–208) and other medical or external deaths (except deaths directly attributed to diabetes, including acute diabetic crises—ie, 250).

### Data analysis

We estimated the sex-specific prevalence of diabetes separately by age and by levels of other vascular risk factors (eg, BMI, cholesterol, blood pressure). We used Cox regression stratified by study (which yields an approximately inverse-variance weighted average of the log death rate ratio [RR] from each study) to estimate the relevance of diabetes to cause-specific mortality, yielding death RRs over the average study period, adjusted for (or stratified by) age at risk (within 5-year age bands from 35–39 to 85–89 years), sex, BMI, systolic and diastolic blood pressure, total cholesterol, and smoking status (classified into three categories: never smoked any type of tobacco regularly, current cigarette smoker, and former or other—including ex-smoker of any type of tobacco, current smoker of pipe or cigar, or smoking status not known). We assessed heterogeneity using Cochran's Q statistic.

We then estimated the absolute occlusive vascular mortality rates in 12 groups defined by diabetes (yes or no), sex (male or female), and age group at risk (30–59, 60–69, or 70–89 years) using Poisson regression to standardise to the average levels of all confounders and to the information-weighted average absolute occlusive vascular mortality rates seen across the different studies. We then calculated the difference in these confounder-adjusted rates (between participants with and without diabetes) by age and sex, along with their 95% CIs. All rates are reported as percentage per year.

To determine whether the relevance of systolic blood pressure, total cholesterol, or BMI to occlusive vascular mortality risk differed between participants with and without diabetes, we divided participants into groups defined by diabetes status and then by levels of each risk factor (six groups). After adjustment for age, sex, study, smoking status, and the other two risk factors not being directly assessed, occlusive vascular death RRs were estimated for each group relative to the group with the lowest level of the risk factor in participants without diabetes (ie, the reference group). We plotted these death RRs against usual (ie, long-term average) systolic blood pressure and total cholesterol^11^, ^12^ and against baseline BMI (since there was little evidence of regression to the mean for BMI in these data).^13^ 95% CIs were calculated using the variance of the log risk, which ascribes an appropriate variance to the log of the death RR in every group (including the reference group).^14^ We did sensitivity analyses including participants excluded for previous cardiovascular disease, and also after excluding studies of only men or only women, and studies that contributed the most to between-study heterogeneity.

Analyses were done with SAS version 9.3 and R version 2.11.1.

### Role of the funding source

The funders of the study had no role in study design, data collection, data analysis, data interpretation, or writing of the report. The corresponding author had full access to all the data in the study and had final responsibility for the decision to submit for publication.

## Results

From 68 prospective studies in the Prospective Studies Collaboration and Asia Pacific Cohort Studies Collaboration studies database, individual participant-level data for 1 017 259 participants were obtained, and 980 793 adults (568 525 [58·0%] men *vs* 412 268 [42·0%] women) were eligible for analyses (8697 were excluded for a history of ischaemic heart disease or stroke and 27 769 for having incomplete information on baseline covariates). The studies were done in 19 countries, and included 516 780 (52·6%) participants from western and central Europe, 271 290 (27·7%) from southeast Asia, 122 330 [12·5%] from North America, and 70 393 (7·2%) from Australasia (appendix). Mean age at recruitment was 46 years (SD 10) for the analysed participants and mean age at occlusive vascular death in the 19 686 participants who died from these causes between ages 35 and 89 years was 66 years (SD 11).

Overall, 42 451 (4·3%) of 980 793 participants reported having diabetes at recruitment (28 450 [5·0%] of 568 525 men, and 14 001 [3·4%] of 412 268 women; appendix). The prevalence of diabetes increased with age, from 2·1% in men and 1·4% in women at age 40 years, to 8·9% in men and 6·0% in women by age 70 years (appendix). Of the 58 700 participants who reported no history of diabetes but who had a measurement of glucose, only 1174 (2·0%) had a glucose measurement that indicated they had undiagnosed diabetes and were counted as having diabetes in the main analyses.

The prevalence of diabetes increased with increasing BMI, systolic blood pressure (adjusted for BMI), and total cholesterol (adjusted for BMI), and decreased with increasing HDL cholesterol (HDL cholesterol was assessed in a subset of 168 341 participants). The prevalence of diabetes was broadly similar irrespective of smoking status (appendix).

**Figure 1:**
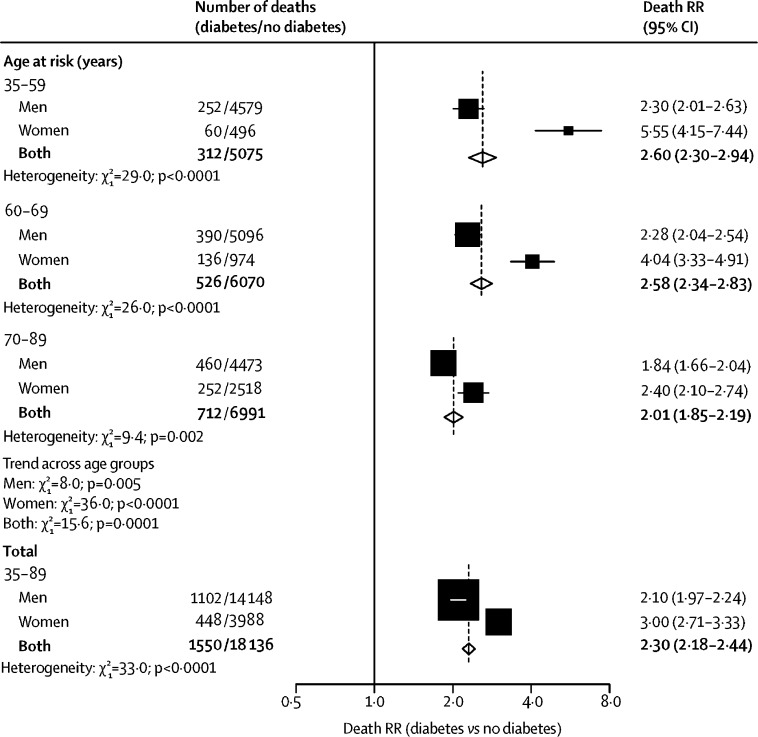
Age-specific and sex-specific relevance of diabetes at study recruitment to occlusive vascular mortality Analyses are stratified by study and adjusted for age at risk, BMI, systolic and diastolic blood pressure, total cholesterol, and smoking status. Each diamond is the inverse-variance weighted average of the two estimates for men and women. The size of the blocks reflects the amount of statistical information (ie, the inverse-variance of the log death RR). RR=rate ratio.

**Figure 2:**
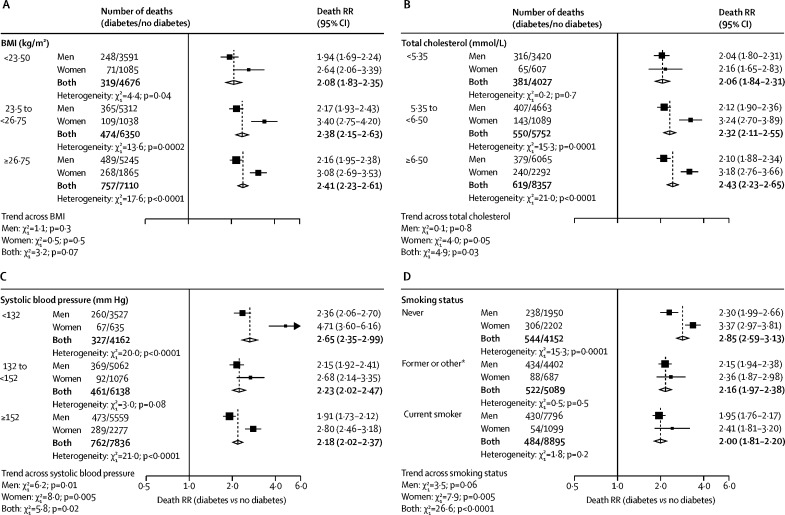
Sex-specific relevance of diabetes at study recruitment to occlusive vascular mortality at ages 35–89 years, by baseline BMI (A), total cholesterol (B), systolic blood pressure (C), and smoking status (D) Each risk factor was split into three strata; approximate thirds for the range of values for BMI, total cholesterol, and systolic blood pressure, and three categories for smoking status. Analyses are stratified by study and adjusted for age at risk, BMI, systolic and diastolic blood pressure, total cholesterol, and smoking status (apart from the analyses stratified by smoking status). Each diamond is the inverse-variance weighted average of the two estimates for men and women. The size of the block reflects the amount of statistical information (ie, the inverse-variance of the log death RR). RR=rate ratio. *Includes ex-smokers of any type of tobacco, current smokers of other types of tobacco, or smoking status not known.

During 9·8 million person-years of follow-up, in participants who were aged between 35 and 89 years, 76 765 participants died, including 19 686 (25·6%) from occlusive vascular causes, of whom 17 919 (91·0%) died from ischaemic heart disease, 1499 (7·6%) from ischaemic stroke, and 268 (1·4%) from other atherosclerotic diseases. Figure 1 summarises the adjusted age-specific and sex-specific death RRs (participants with diabetes *vs* those without) for occlusive vascular mortality (stratified by study and adjusted for age at risk, BMI, systolic and diastolic blood pressure, total cholesterol, and smoking status). Overall, at ages 35–89 years, diabetes more than doubled occlusive vascular mortality risk (death RR 2·30, 95% CI 2·18–2·44), but the death RR was higher for women (3·00, 2·71–3·33) than for men (2·10, 1·97–2·24). The death RRs were higher at younger ages than at older ages (figure 1; p=0·0001 for trend across age groups). This finding was especially apparent among women, for whom the diabetes death RR was 5·55 (95% CI 4·15–7·44) at ages 35–59 years. These higher death RRs in women largely persisted within levels of other vascular risk factors (figure 2). When we did analyses separately for ischaemic heart disease and ischaemic stroke, the age-specific and sex-specific death RRs were similar to those seen for all occlusive vascular deaths considered together (p<0·05 for the heterogeneity test between men and women for overall death RRs for both ischaemic heart disease and ischaemic stroke; appendix); we did not analyse deaths from other specific atherosclerotic diseases because small numbers precluded reliable analyses. The results were not materially changed after excluding studies that recruited only men or only women (appendix), or when excluding the studies that contributed most to between-study heterogeneity (data not shown). Sex differences in death RRs were also evident among participants who reported a history of ischaemic heart disease or stroke (appendix) and separately within each of the three studied regions (ie, Europe, the USA or Australasia, and Asia; appendix).

The underlying confounder-adjusted occlusive vascular mortality rates at any given age were higher among men than women; therefore, the age-specific absolute excess occlusive vascular mortality risk associated with diabetes was similar for men and women (table). At ages 35–59 years, the adjusted excess mortality associated with diabetes was 0·05% (95% CI 0·03–0·07) per year in women compared with 0·08% (0·05–0·10) per year in men; the corresponding excess mortality at ages 70–89 years was 1·08% (0·84–1·32) per year in women and 0·91% (0·77–1·05) per year in men.

**Table:** Adjusted rates of occlusive vascular death among people with and without diabetes at recruitment, by age and sex

	**Diabetes**			**No diabetes**			**Rate difference (95% CI)**
	Deaths (n)	Person-years	Adjusted rate*	Deaths (n)	Person-years	Adjusted rate*	
**Aged 35–59 years**							
Men	252	151 321	0·13%	4579	4 168 660	0·06%	0·08% (0·05–0·10)
Women	60	72 756	0·06%	496	2 878 295	0·01%	0·05% (0·03–0·07)
**Aged 60–69 years**							
Men	390	62 605	0·52%	5096	1 062 761	0·24%	0·28% (0·22–0·34)
Women	136	32 547	0·28%	974	720 144	0·07%	0·20% (0·13–0·27)
**Aged 70–89 years**							
Men	460	30 489	2·05%	4473	350 754	1·14%	0·91% (0·77–1·05)
Women	252	13 001	1·92%	2518	267 099	0·84%	1·08% (0·84–1·32)

Data are n, person-years, or percentage per year, with 95% CIs in parentheses where stated.

* Absolute rates are estimated by use of Poisson regression stratified by study and adjusted for age at risk (in 5-year age groups), BMI, systolic and diastolic blood pressure, total cholesterol, and smoking status.

**Figure 3:**
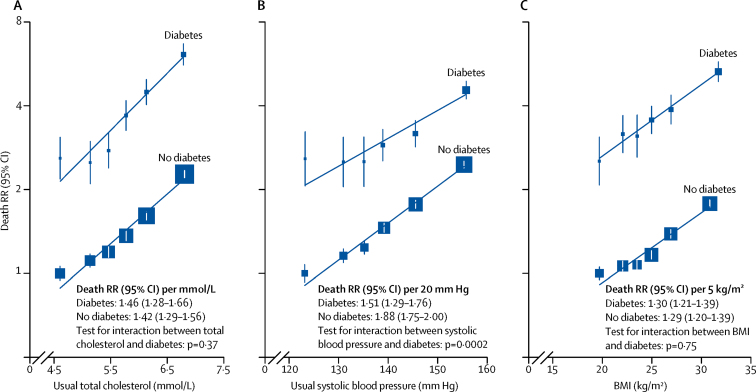
Relevance of total cholesterol (A), systolic blood pressure (B), and BMI (C) to RR of occlusive vascular mortality at ages 35–89 years, by diabetes status at study recruitment Analyses are stratified by study and sex, and adjusted for age at risk, smoking status, and, if appropriate, total cholesterol, systolic and diastolic blood pressure, and BMI. Usual total cholesterol and systolic blood pressure are the long-term average level of that risk factor. Regression dilution ratios of 0·65 for total cholesterol and 0·67 for systolic blood pressure were calculated by regressing serial measurements from 175 000 participants with at least one re-measurement, on average, 3 years later, on baseline levels of these risk factors. No such adjustment was applied for BMI, since one single measurement at baseline was highly correlated with long-term BMI. The vertical lines through the plotted boxes are 95% CIs that reflect only the variance of the log risk in that group (and are therefore shown for every group including the reference group), and the size of each box reflects the amount of statistical information. RR=rate ratio.

For usual (ie, long-term average level) total cholesterol, usual systolic blood pressure, and baseline BMI, log-linear positive associations with occlusive vascular mortality risk were seen both among participants with and without diabetes (figure 3). For total cholesterol and BMI, the strength of the associations was similar for participants with and those without diabetes, but for systolic blood pressure the association was somewhat weaker among those with diabetes than among those without diabetes. However, because the absolute risk of occlusive vascular mortality was appreciably higher in participants with diabetes, the crude absolute risks associated with higher levels of all three of these vascular risk factors were much greater among participants with diabetes than those without. These findings were similar when men and women were considered separately (appendix).

**Figure 4:**
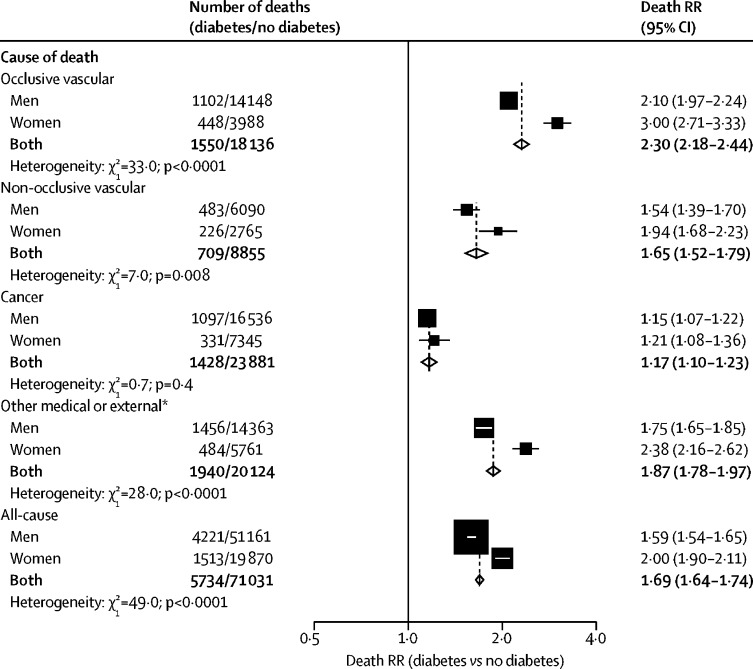
Sex-specific relevance of diabetes at study recruitment to cause-specific and all-cause mortality at ages 35–89 years Analyses are stratified by study and adjusted for age at risk, BMI, systolic and diastolic blood pressure, total cholesterol, and smoking status. Each diamond is the inverse-variance weighted average of the two estimates for men and women. The size of the block reflects the amount of statistical information (ie, the inverse-variance of the log death RR). RR=rate ratio. *Except for deaths attributed directly to diabetes, including acute diabetic crises (ICD-9 code 250), which occurred among 107 of the participants with diabetes at recruitment and 35 of the participants without diabetes at recruitment.

For non-occlusive vascular mortality, the death RRs comparing individuals with and without diabetes were somewhat weaker than for occlusive vascular mortality, but, as for occlusive vascular mortality, death RRs were larger for women than for men (figure 4). The death RRs for cancer mortality were considerably smaller (overall death RR 1·17, 95% CI 1·10–1·23) than for vascular causes, and similar among men and women. Diabetes was associated with an approximate doubling in the risk of death from the composite of any other medical or external cause (death RR 1·87, 1·78–1·97; excluding 142 deaths attributed directly to diabetes).

## Discussion

In this analysis of individual participant-level data from nearly 1 million adults with no previous vascular disease who were followed up prospectively to monitor mortality, we determined that having diabetes at recruitment was associated with a doubling in death rates from ischaemic heart disease and ischaemic stroke among men, but a tripling of these death rates among women. For both men and women, the death RRs for occlusive vascular mortality were greater in early middle-aged (ie, 35–59 years) than in older patients, so that diabetes was associated with an almost six times higher occlusive vascular death rate among women aged 35–59 years, even after controlling for other established major vascular risk factors.

At any given age, the underlying confounder-adjusted occlusive vascular death rates were higher for men than for women, so despite higher death RRs among women than among men, we estimate that the absolute excess risk of occlusive vascular mortality associated with diabetes was similar in men and women. The adjusted occlusive vascular mortality rates among women with diabetes aged 35–59 or 60–69 years were similar to those among men without diabetes of a similar age (table).

Sex differences in the relative excess of occlusive vascular risk associated with diabetes have been reported in several studies,^3^, ^4^, ^15^, ^16^ but most have not considered how this relative excess risk translates to absolute excess risk. By contrast with these studies, the main strength of our analysis is the inclusion of studies with complete availability of baseline information on systolic and diastolic blood pressure, total cholesterol, BMI, and smoking status in a large number of apparently healthy people (ie, without occlusive vascular disease) followed up prospectively. Underprescription of cardiovascular-risk-modifying therapy among women has been described,^17^ but since many of the cohort studies included in our meta-analysis were completed before the introduction of statin therapy and widespread screening for high blood pressure for primary prevention, our findings suggest that underprescription among women is unlikely to account for the differences between the sexes in our calculated diabetes death RRs. Indeed, our findings remain relevant, since results from a study of 1·9 million patients in an English primary-care database who were identified and followed up between 1998 and 2010 showed that relative risks for non-fatal ischaemic heart disease were somewhat higher among women than among men, even after adjustment for disease management via antihypertensive medication and statin use^16^ (which are similarly efficacious in both sexes^18^, ^19^). However, sex differences in the management of cardiac disease during and after treatment in hospital might, to some extent, help to account for the higher diabetes death RRs among women than among men.^20^

Several hypotheses that could account for sex differences in diabetes-associated vascular risk remain unexplored. Hormone-replacement therapy has been shown to increase cardiovascular risk,^21^ and both oestrogens and androgens can affect lipid metabolism;^22^ therefore, future analyses considering sex-hormone concentrations and subclasses of lipids would be of interest. 23, 24, 25 Sex differences in inflammation might also exist,^26^ and since targeting interleukin-1β can reduce occlusive vascular risk,^27^ inflammation should be a focus of future research aimed at devising novel therapeutic approaches.

The main limitation of this study was the absence of phenotypic information on diabetes and its complications, which have been shown to affect risk (eg, type of diabetes, glycaemic control,^3^ duration of diabetes,^28^ and the presence of complications such as renal disease^29^). Other studies have considered these factors, but rarely are all factors comprehensively measured, and whether sex differences in diabetes phenotype could account for sex differences in the relation of diabetes with occlusive vascular mortality risk is unclear. For example, type 1 diabetes might be associated with high cardiovascular risk,^30^ but among adults with diabetes the proportion who have type 1 diabetes is usually low in most populations (3–15%) and, if anything, this proportion is higher in men than in women.^31^ Differences in glycaemic control are also not a clear explanation for sex differences observed in the English primary care study of 1·9 million patients,^16^ since diabetes was associated with a somewhat higher risk of ischaemic heart disease in women than in men despite similar prescription of diabetes treatments, and glycaemic control being, if anything, slightly better in women than in men.^16^ However, in a prospective study of 150 000 Hispanic adults from Mexico City, Mexico,^5^ no difference was seen between men and women in the diabetes death RR for vascular causes (or for all causes combined). Notably, both men and women in that study had a similar duration of diabetes and similarly poor glycaemic control. Additionally, our meta-analysis does not take into account the effects of undiagnosed diabetes at baseline or the development of new diabetes during follow-up. However, among participants with glucose measurements, only 2% of those not reporting a diagnosis at baseline had measurements that indicated the presence of undiagnosed diabetes, and, therefore, if a similarly small percentage of participants developed diabetes during follow-up then the reported death RRs would not be greatly affected since these people would contribute only a very small proportion of those classified as not having diabetes at baseline. Another limitation of this study is that the cohorts were recruited some years ago, with baseline data ranging from 1949 to 1997. Although this aspect of the study also has some advantages (primarily that the participants were recruited before widespread treatment with statins or blood-pressure-lowering medication), we must recognise that treatment of both diabetes and vascular risk factors have changed substantially over the past few decades and these changes might affect the current vascular risks associated with a diagnosis of diabetes. Hence, analysis of recently established and future prospective cohorts is needed to assess whether developments in the treatment of both diabetes and vascular risk factors over the past few decades are having any effect on contemporary diabetes-associated risks and on the age and sex differences in these associations.

Our analyses showed that the strength of the association of total cholesterol and BMI with occlusive vascular mortality risk was similar irrespective of the presence of diabetes, and that at any level of these risk factors, diabetes carries a substantially increased risk (figure 3). Hence, the absolute relevance of these risk factors to occlusive vascular disease would be greater for people with diabetes than those without. Although the association between systolic blood pressure and occlusive vascular mortality risk was slightly weaker among participants with diabetes, the absolute relevance to risk would again be greater among those with diabetes than those without diabetes.

In the WHO 2013–20 non-communicable disease global plan targets,^32^ one of the main priorities is a 25% reduction in the risk of premature mortality from cardiovascular diseases and diabetes by 2025 relative to 2013. The continuous nature of the associations of conventional risk factors with vascular events among people with diabetes in our analyses supports data from randomised trials^33^, ^34^ that suggest that the vascular benefits of intensive versus recommended standard blood-pressure-lowering medications and targets are similar among those with and without diabetes, and support the existing guideline recommendation^35^ favouring treatment of absolute risk of disease rather than threshold concentrations of cholesterol. Since the relevance of blood pressure, blood cholesterol, and BMI to occlusive vascular mortality risk was broadly similar among people with and without diabetes, the absolute benefits of strategies to achieve effective lowering of these multiple risk factors in both women and men with diabetes will help to achieve global health priorities. However, the use of only these strategies would not be expected to reduce all the relative excess of diabetes-associated vascular risk seen among women, since this excess risk does not seem to be explained by these traditional major vascular risk factors.

Correspondence to: Dr Sarah Lewington, MRC Population Health Research Unit, Clinical Trial Service Unit and Epidemiological Studies Unit (CTSU), Nuffield Department of Population Health, University of Oxford, Oxford OX3 7LF, UK **sarah.lewington@ndph.ox.ac.uk**
