# Supplementary material for: Sex-specific relevance of diabetes to occlusive vascular and other mortality: a collaborative meta-analysis of individual data from 980 793 adults from 68 prospective studies
Source: Lancet Diabetes Endocrinol. 2018 Jul;6(7):538–46. doi: 10.1016/S2213-8587(18)30079-2 (PMC6008496; doi:10.1016/S2213-8587(18)30079-2)
Supplement: Supplementary appendix [file mmc1.pdf]

THE LANCET
Diabetes & Endocrinology

Supplementary appendix

This appendix formed part of the original submission and has been peer reviewed. We post it as supplied by the authors.

Supplement to: Prospective Studies Collaboration and Asia Pacific Cohort Studies Collaboration. Sex-specific relevance of diabetes to occlusive vascular and other mortality: a collaborative meta-analysis of individual data from 980 793 adults from 68 prospective studies. *Lancet Diabetes Endocrinol* 2018; published online May 8. http://dx.doi.org/10.1016/S2213-8587(18)30079-2.

Sex differences in diabetes-associated mortality: Meta-analysis of 20,000 occlusive vascular deaths in 1 million adults

Supplementary Appendix, Table of Contents

Webtable 1:	Study characteristics and references	Pages 3-11
Webtable 2:	Baseline characteristics of men and women with and without diabetes at study recruitment	Page 12
Webtable 3:	Relevance of: (a) total cholesterol; (b) SBP; and (c) BMI to occlusive vascular mortality at ages 35-89 years, by sex and diabetes at study recruitment	Page 13
Webfigure 1:	Sex-specific prevalence of diabetes at study recruitment by baseline characteristics	Page 14
Webfigure 2:	Age and sex-specific relevance of diabetes at study recruitment to IHD and ischaemic stroke mortality	Page 15
Webfigure 3:	Age and sex-specific relevance of diabetes at study recruitment to occlusive vascular mortality (limited to studies that included both men and women)	Page 16
Webfigure 4:	Relevance of diabetes at study recruitment to occlusive vascular mortality, by age and sex in those with prior IHD or stroke	Page 17
Webfigure 5	Relevance of diabetes at study recruitment to occlusive vascular mortality at ages 35-89, by sex and region	Page 18

Webtable 1: Study characteristics

	Median year of screening	No. participants	No. occlusive vascular deaths	Men, %	Mean age (years) at: Screening	Mean age (years) at: Occlusive vascular death	Diabetes, %	Mean (SD) BMI, kg/m^2	Mean (SD) SBP, mmHg	Mean (SD) DBP, mmHg	Mean (SD) Total cholesterol, mmol/L	Mean (SD) HDL cholesterol, mmol/L	Current smoker, %
**Europe**													
Belgian Inter-university Research on Nutrition & Health (BIRNH)	1983	10027	131	52	48	70	2.0	25.9 (4.0)	134 (19)	81 (12)	6.1 (1.2)	1.4 (0.4)	---
British Regional Heart Study (BRHS)	1979	7347	443	100	50	62	1.2	25.5 (3.2)	145 (21)	82 (13)	6.3 (1.0)	---	41
British United Provident Association (BUPA)	1981	5235	95	100	46	62	0.9	25.0 (2.7)	130 (16)	82 (11)	6.2 (1.2)	1.3 (0.4)	21
Caerphilly and Speedwell	1981	1750	99	100	52	61	1.7	26.0 (3.5)	140 (18)	88 (12)	5.7 (1.1)	1.1 (0.3)	44
CB project	1977	48652	239	48	39	51	0.8	24.6 (3.4)	130 (17)	79 (11)	5.4 (1.1)	---	52
Centre d'Investigations Preventives et Cliniques (IPC)	1977	179090	381	57	41	62	5.9	23.8 (3.4)	131 (14)	80 (10)	5.6 (1.1)	---	30
Copenhagen City Heart Study	1977	12575	519	45	52	69	1.7	25.1 (4.1)	137 (22)	83 (12)	6.1 (1.2)	1.5 (0.4)	59
Finish Mobile Clinic Survey	---	39098	3522	54	41	69	1.5	25.2 (3.8)	142 (23)	80 (14)	6.6 (1.4)	---	34
FINRISK	1983	37009	1853	48	43	63	2.5	26.1 (4.2)	143 (21)	87 (12)	6.4 (1.3)	1.4 (0.4)	31
Glostrup Population Studies	1969	9031	185	50	48	70	2.0	24.8 (4.0)	127 (19)	79 (11)	6.1 (1.2)	1.5 (0.4)	52
Gothenburg Prospective Study of Women	1963	1400	69	0	47	70	0.8	24.0 (3.7)	133 (22)	85 (11)	6.8 (1.2)	---	41
Israeli Ischaemic Heart Disease Study	---	9687	942	100	49	67	5.5	25.7 (3.3)	135 (20)	84 (11)	5.4 (1.0)	0.9 (0.2)	51
Norwegian Counties Study	1975	46738	908	50	42	54	0.6	24.9 (3.6)	134 (17)	83 (11)	6.3 (1.3)	---	42
Northwick Park Heart Study (NPHS)	1973	3034	125	71	46	67	0.0	24.9 (3.2)	138 (22)	85 (14)	6.0 (1.2)	---	38
Oslo	1970	15683	762	100	45	57	0.9	24.7 (2.9)	136 (16)	87 (11)	6.9 (1.3)	---	46
Paris Prospective Study	1982	7455	212	100	47	64	2.7	25.9 (3.3)	141 (21)	80 (14)	5.8 (1.1)	---	74
Prospective Cardiovascular Munster Study (PROCAM)	1983	13578	101	76	47	58	4.5	26.0 (3.4)	131 (19)	85 (11)	5.8 (1.1)	1.3 (0.4)	30
Renfrew/Paisley	1975	10202	835	45	54	67	1.1	25.6 (3.8)	148 (23)	85 (13)	6.1 (1.1)	---	51
Scottish Heart Health Study (SHHS)	1986	11117	170	50	49	59	1.3	25.7 (4.0)	132 (20)	82 (12)	6.4 (1.3)	1.5 (0.4)	36
Caerphilly and Speedwell	1980	1786	101	100	54	64	1.5	25.5 (3.1)	138 (22)	86 (13)	5.9 (1.2)	1.1 (0.4)	38
Tromso	1979	14339	102	52	36	54	0.4	23.7 (3.2)	126 (15)	81 (10)	5.9 (1.2)	1.6 (0.5)	---
United Kingdom Heart Disease Prevention Project (UKHDPP)	1971	8724	1033	100	49	65	1.1	25.4 (3.2)	139 (19)	84 (12)	5.6 (1.0)	---	51
Whitehall	1968	18144	1340	100	52	64	0.9	24.7 (3.0)	136 (21)	85 (14)	5.1 (1.2)	---	42
West Scotland	1971	5079	533	86	47	64	0.6	25.0 (3.2)	134 (17)	83 (10)	5.8 (1.0)	---	56
**Subtotal**	**1980**	**516780**	**14700**	**61**	**43**	**65**	**3.0**	**24.7 (3.6)**	**134 (19)**	**82 (12)**	**5.9 (1.3)**	**1.4 (0.4)**	**38**

	Median year of screening	No. participants	No. occlusive vascular deaths	Men, %	Mean age (years) at: Screening	Occulsive vascular death	Diabetes, %	Mean (SD) BMI, kg/m^2	SBP, mmHg	DBP, mmHg	LDL cholesterol, mmol/L	HDL cholesterol, mmol/L	Current smoker, %
**North America/Australasia**													
Atherosclerosis Risk in Communities (ARIC)	1988	14484	130	44	54	61	6.7	27.5 (5.1)	121 (19)	74 (11)	5.5 (1.1)	1.3 (0.4)	26
Australian Longitudinal Study of Aging	1992	883	37	49	76	82	7.6	25.9 (4.0)	149 (22)	80 (11)	5.9 (1.2)	1.3 (0.4)	9
The Australian Risk Factor Prevalence Study	1989	7705	50	49	45	66	1.7	25.5 (4.2)	126 (18)	79 (11)	5.6 (1.1)	1.3 (0.4)	23
Busselton Health Study	1972	2736	161	47	41	76	1.4	24.5 (3.7)	130 (20)	74 (13)	5.7 (1.3)	1.5 (0.4)	30
Cardiovascular Health Study	1989	4339	90	40	72	79	9.7	26.4 (4.6)	136 (21)	70 (11)	5.6 (1.0)	1.4 (0.4)	12
Charleston	1961	1964	407	45	49	72	3.0	25.4 (4.8)	145 (29)	86 (12)	6.1 (1.3)	---	47
Fletcher Challenge	1993	8338	75	73	47	69	2.7	26.7 (4.1)	127 (17)	78 (11)	5.5 (1.1)	---	22
Framingham Heart Study	1951	2948	438	48	43	70	1.3	25.5 (4.2)	138 (24)	86 (13)	5.7 (1.1)	---	63
Honolulu Heart Program	1967	7137	511	100	54	73	25.4	23.9 (3.1)	133 (21)	82 (12)	5.6 (1.0)	---	45
Melbourne Collaborative Cohort	1993	38980	221	40	54	67	4.9	26.8 (4.4)	137 (20)	77 (12)	5.5 (1.1)	---	11
Minnesota Heart Survey	1986	6146	27	47	47	66	4.5	26.8 (4.9)	122 (17)	75 (11)	5.3 (1.0)	1.3 (0.4)	28
Newcastle	1989	3060	24	49	53	67	2.5	27.0 (4.6)	133 (20)	79 (11)	5.8 (1.1)	1.3 (0.4)	19
First National Health & Nutrition Examination Survey Epidemiologic Follow-up Study (NHEFS)	1972	9721	894	39	49	75	5.2	25.6 (4.9)	135 (24)	83 (13)	5.7 (1.3)	---	37
Perth	1983	8691	121	53	46	69	1.8	25.2 (3.9)	130 (20)	81 (11)	5.8 (1.2)	1.4 (0.4)	---
Puerto Rico Health Heart Program	1973	4624	451	45	55	78	3.8	24.5 (3.5)	131 (23)	79 (11)	5.4 (1.0)	---	26
Tecumseh	1960	3335	424	49	47	70	9.4	25.8 (4.6)	139 (22)	84 (12)	5.4 (1.0)	---	---
US Health Professionals Follow-up Study	1986	21594	140	100	54	66	3.2	25.4 (3.1)	129 (13)	81 (7)	5.2 (0.9)	---	9
US Nurses' Health Study (NHS II)	1988	46038	80	0	55	64	4.6	25.6 (4.8)	126 (13)	79 (9)	5.3 (1.2)	---	16
**Subtotal**	**1988**	**192723**	**4281**	**43**	**53**	**72**	**5.2**	**26.0 (4.4)**	**130 (19)**	**79 (11)**	**5.5 (1.1)**	**1.3 (0.4)**	**18**

	Median year of screening	No. participants	No. occlusive vascular deaths	Men, %	Mean age (years) at:		Diabetes, %	Mean (SD)					Current smoker, %
					Screening	Occlusive vascular death		BMI, kg/m^2	SBP, mmHg	DBP, mmHg	Total cholesterol, mmol/L	HDL cholesterol, mmol/L	
**Asia**													
Aito Town	1981	1560	10	44	51	70	2.6	22.6 (3.0)	136 (21)	79 (13)	4.6 (0.9)	1.4 (0.4)	19
Akabane	1985	1803	13	44	54	67	2.4	22.5 (3.0)	125 (19)	74 (12)	5.0 (0.9)	1.2 (0.3)	28
Anzhen 2	1992	4139	1	49	47	50	10.7	24.0 (3.3)	122 (18)	78 (11)	4.7 (0.9)	1.4 (0.4)	21
Beijing Ageing	1992	1390	0	51	69	.	26.5	23.2 (3.9)	141 (25)	81 (13)	4.3 (1.0)	1.5 (0.6)	32
Civil Serivce Workers	1991	9147	2	67	47	56	1.7	22.5 (2.7)	126 (18)	75 (11)	5.2 (0.9)	1.4 (0.4)	38
Electricity Generating Authority of Thailand (EGAT)	1985	3488	28	77	43	53	2.8	23.1 (3.1)	121 (16)	75 (11)	5.8 (1.1)	1.2 (0.3)	43
Fangshan Cohorts	1992	806	0	33	47	.	6.7	25.0 (3.7)	133 (25)	79 (12)	4.6 (1.1)	1.4 (0.4)	39
Guangzhou Occupational Cohort	1991	1847	2	71	45	64	8.4	22.8 (2.9)	115 (16)	76 (10)	5.5 (1.3)	1.3 (0.4)	46
Hong Kong	1991	123	2	48	77	87	13.0	22.4 (3.6)	148 (21)	80 (12)	5.4 (0.9)	1.6 (0.5)	18
Ikawa	1977	2098	31	44	52	72	1.2	23.6 (3.2)	137 (22)	83 (12)	4.7 (0.8)	---	31
Japan Railways	1978	24708	32	100	43	54	1.9	22.8 (2.7)	129 (17)	81 (12)	4.6 (0.9)	---	65
Korean Medical Insurance Corporation (KMIC)	1992	183349	164	63	44	51	7.2	23.0 (2.5)	122 (15)	80 (10)	5.0 (0.9)	---	9
Kounan	1991	1031	7	45	53	72	11.9	21.9 (2.9)	130 (19)	78 (11)	4.9 (0.9)	1.6 (0.4)	31
Kyowa	1983	4099	25	43	54	69	4.3	23.5 (3.3)	137 (20)	81 (12)	4.9 (1.0)	---	33
Miyama	1989	408	0	38	59	.	2.5	22.3 (3.0)	129 (23)	77 (12)	5.1 (0.9)	1.3 (0.3)	24
Noichi	1976	2179	32	37	54	72	5.0	22.9 (3.2)	136 (21)	80 (12)	4.8 (0.9)	---	26
Ohasama	1992	1787	4	35	58	78	9.5	23.3 (3.1)	127 (17)	72 (12)	5.0 (0.9)	1.4 (0.4)	21
Saitama	1987	3429	43	38	54	76	1.7	22.4 (2.9)	135 (20)	80 (12)	5.0 (1.0)	---	29
Shibata	1977	2223	133	43	56	78	1.1	22.4 (3.0)	130 (21)	78 (12)	4.6 (1.2)	---	33
Shigaraki Town	1993	3301	5	41	57	78	6.8	22.5 (3.0)	132 (19)	78 (12)	5.0 (0.9)	1.4 (0.4)	29
Shirakawa	1977	4394	74	46	49	69	0.9	21.5 (2.7)	127 (22)	77 (13)	4.7 (0.9)	---	35
Singapore Heart & Thyroid	1984	2152	24	50	41	66	10.8	23.6 (4.3)	123 (21)	76 (12)	5.9 (1.2)	0.9 (0.4)	22
Singapore National Health Survey 1992 (NHS92)	1992	2495	20	48	43	59	11.9	23.7 (4.1)	121 (19)	71 (12)	5.5 (1.0)	1.2 (0.3)	18
Tanno/Soubetsu	1977	1972	30	47	51	65	7.0	23.6 (3.2)	133 (20)	83 (10)	4.9 (1.0)	---	38
Two Township Study in Taiwan (CVDFACTS)	1991	4736	17	45	50	70	2.8	23.8 (3.3)	120 (19)	76 (11)	5.0 (1.2)	1.2 (0.4)	22
Yunnan Tin Miner	1992	2626	6	96	54	67	0.5	21.9 (2.9)	122 (21)	80 (13)	4.3 (0.8)	1.4 (0.4)	69
**Subtotal**	**1992**	**271290**	**705**	**64**	**45**	**66**	**6.2**	**23.0 (2.7)**	**124 (17)**	**79 (11)**	**4.9 (0.9)**	**1.3 (0.4)**	**20**
**Total**	**1985**	**980793**	**19686**	**58**	**46**	**66**	**4.3**	**24.5 (3.7)**	**131 (19)**	**81 (11)**	**5.5 (1.2)**	**1.4 (0.4)**	**29**

References to studies in Webtable 1 by region

EUROPE: 24 studies

Belgian Inter-university Research on Nutrition and Health (BIRNH): De Backer G. Nutrition and Health: an interuniversity study. Regional differences in dietary habits, coronary risk factors and mortality rates in Belgium. I. Design and methodology. *Acta Cardiol* 1989; 94: 89–99.

British Regional Heart Study (BRHS): Shaper AG, Pocock SJ, Walker M, Cohen NM, Wale CJ, Thomson AG. British Regional Heart Study: cardiovascular risk factors in middle-aged men in 24 towns. *BMJ* 1981; 283: 179–86.

British United Provident Association (BUPA) Study: Law MR, Wald NJ, Hackshaw A, Bailey A. Systematic underestimation of association between serum cholesterol concentration and ischaemic heart disease in observational studies: data from the BUPA study. *BMJ* 1994; 308: 363–6.

Caerphilly and Speedwell Studies: The Caerphilly and Speedwell Collaborative Group. Caerphilly and Speedwell collaborative heart disease studies. *J Epidemiol Community Health* 1984; 38: 259–62.

CB project: Verschuren MWM, Kromhout D. Total cholesterol concentration and mortality at a relatively young age: do men and women differ? *BMJ* 1995; 311: 779–83.

Centre d'Investigations Preventives et Cliniques (IPC): Benetos A, Rudnichi A, Thomas F, Safar M, Guize L. Influence of heart rate on mortality in a French population: role of age, gender, and blood pressure. *Hypertension* 1999 Jan; 33(1): 44–52.

Copenhagen City Heart Study: Schnohr P, Jensen G, Lange P, Scharling H, Appleyard M. The Copenhagen City Heart Study. Osterbroundersogelsen. A book of tables with data from the third examination (1991–94). *Eur Heart J* 2001; 3 (suppl): 1–83.

Finnish Mobile Clinic Survey: Reunanen A, Aromaa A, Pyorala K, Punsar S, Maatela J, Knekt P. The Social Insurance Institution's coronary heart disease study: baseline data and 5-year mortality experience. *Acta Med Scand* 1983; 673 (suppl): 1–120.

FINRISK: Antikainen RL, Jousilahti P, Vanhanen H, Tuomilehto J. Excess mortality associated with increased pulse pressure among middle-aged men and women is explained by high systolic blood pressure. *J Hypertens* 2002; 18: 417–23.

Glostrup Population Studies: Osler M, Linneberg A, Glümer C, Jørgensen T. The cohorts at the Research Centre for Prevention and Health, formerly 'The Glostrup Population Studies'. *Int J Epidemiol.* 2011 Jun; 40(3): 602–10.

Gothenburg Prospective Study of Women: Bengtsson C, Gredmark T, Hallberg L, et al. The population study of women in Gothenburg 1980–81—the third phase of a longitudinal study. Comparison between participants and nonparticipants. *Scand J Soc Med* 1989; 17: 141–5.

Israeli Ischaemic Heart Disease Study: Goldbourt U, Yaari S, Medalie JH. Factors predictive of long-term coronary heart disease mortality among 10 059 male Israeli civil servants and municipal employees: a 23-year mortality follow-up in the Israeli Ischemic Heart Disease Study. *Cardiology* 1993; 82: 100–21.

Norwegian Counties Study: Bjartveit K, Foss OP, Gjervig T, Lund-Larsen PG. The cardiovascular disease study in Norwegian counties: background and organization. *Acta Med Scand* 1979; 634 (suppl): 1–70.

Northwick Park Heart Study: Meade TW, Mellows S, Brozovic M, et al. Haemostatic function and ischaemic heart disease: principal results of the Northwick Park Heart Study. *Lancet* 1986; 2: 533–7.

Oslo Study: Holme I, Solberg L. A., Weissfeld L, et al. Coronary risk factors and their pathway of action through coronary raised lesions, coronary stenoses and coronary death: Multivariate statistical analysis of an autopsy series: The Oslo study. *Am. J. Cardiology* 1965; 55: 40–47.

Paris Prospective Study: Filipovsky J, Ducimetiere P, Darne B, Richard JL. Abdominal body mass distribution and elevated blood pressure are associated with increased risk of death from cardiovascular diseases and cancer in middle-aged men: the results of a 15- to 20-year follow-up in the Paris prospective study I. *Int J Obes Relat Metab Disord* 1993; 17: 197–203.

Prospective Cardiovascular Münster (PROCAM) Study: Cullen P, Schulte H, Assmann G. The Munster Heart Study (PROCAM): total mortality in middle-aged men is increased at low total and LDL cholesterol concentrations in smokers but not in nonsmokers. *Circulation* 1997; 96: 2128–36.

Renfrew/Paisley (Midspan) Study: Hawthorne VM, Watt GC, Hart CL, Hole DJ, Davey Smith G, Gillis CR. Cardiorespiratory disease in men and women in urban Scotland: baseline characteristics of the Renfrew/Paisley (midspan) study population. *Scott Med J* 1995; 40: 102–7.

Scottish Heart Health Study (SHHS): Tunstall-Pedoe H, Woodward M, Tavendale R, A'Brook R, McCluskey MK. Comparison of the prediction by 27 different factors of coronary heart disease and death in men and women of the Scottish Heart Health Study: cohort study. *BMJ* 1997; 315: 722–29.

Tromsø Study: Bonaa KH, Thelle DS. Association between blood pressure and serum lipids in a population: the Tromso Study. *Circulation* 1991; 83: 1305–14.

United Kingdom Heart Disease Prevention Project (UK HDDP): Rose G, Tunstall-Pedoe HD, Heller RF. UK heart disease prevention project: incidence and mortality results. *Lancet* 1983; 1: 1062–6.

West Scotland Study: Davey Smith G, Hart C, Hole D, et al. Education and occupational social class: which is the more important indicator of mortality risk? *J Epidemiol Community Health* 1998; 52: 153–60.

Whitehall Study: Reid DD, Brett GZ, Hamilton PJ, Jarrett RJ, Keen H, Rose G. Cardiorespiratory disease and diabetes among middle-aged male civil servants: a study of screening and intervention. *Lancet* 1974; 1: 469–73.

NORTH AMERICA/AUSTRALASIA: 18 studies

Atherosclerosis Risk in Communities (ARIC): The ARIC investigators. The Atherosclerosis Risk in Communities (ARIC) Study: design and objectives. *Am J Epidemiol* 1989; 129: 687–702.

Australian Longitudinal Study of Aging: Andrews G, Cheok F, Carr S. The Australian Longitudinal Study of Ageing. *Australian Journal on Ageing* 1989; 8: 31–5.

The Australian Risk Factor Prevalence Study: Australian Risk Factor Prevalence Study Management Committee. Book Survey No. 3 1989. Canberra: National Heart Foundation of Australia and Australia Institute of Health; 1990.

Busselton Health Study: Knuiman MW, Vu HT, Bartholomew HC. Multivariate risk estimation for coronary heart disease: the Busselton Health Study. *Aust NZ J Public Health* 1998; 22: 747–53.

Cardiovascular Health Study: Fried LP, Borhani NO, Enright P, et al. The Cardiovascular Health Study: design and rationale. *Ann Epidemiol* 1991; 1: 263–76.

Charleston Study: Keil JE, Sutherland SE, Knapp RG, Lackland DT, Gazes PC, Tyroler HA. Mortality rates and risk factors for coronary disease in black as compared with white men and women. *N Engl J Med* 1993; 329: 73–8.

Fletcher Challenge Study: MacMahon S, Norton R, Jackson R, et al. Fletcher Challenge-University of Auckland Heart & Health Study: design and baseline findings. *N Z Med J.* 1995 Dec 8; 108(1013): 499–502.

Framingham Heart Study: Dawber TR, Meadors GF, Moore FE, Jr. Epidemiological approaches to heart disease: the Framingham Study. *Am J Public Health* 1951; 41(3): 279–286.

Honolulu Heart Program: Kagan A, ed. The Honolulu Heart Program: an Epidemiological Study of Coronary Heart Disease and Stroke. Reading, UK: Harwood Academic Publishers, 1996.

Melbourne Collaborative Cohort: Giles GG, English DR. The Melbourne Collaborative Cohort Study. *IARC Sci Publ.* 2002; 156: 69–70.

Minnesota Heart Survey: Luepker RV, Jacobs DR, Shahar E, et al. Ten-year trends (1985 through 1995) in acute coronary heart disease mortality, morbidity and medical care: the Minnesota Heart Survey. *Circulation* 2001; 104: 19–24.

Newcastle Study: Loxton D, Tooth L, Harris ML , et al. Cohort Profile: The Australian Longitudinal Study on Women's Health (ALSWH) 1989-95 cohort*.* *Int J Epidemiol.* 2018 Apr 1; 47(2): 391-2e.

First National Health and Nutrition Examination Survey Epidemiologic Follow-up Study (NHEFS): Cox CS, Mussolino ME, Rothwell ST, et al. Plan and operation of the NHANES I Epidemiologic Followup Study, 1992. National Center for Health Statistics. *Vital Health Stat* 1997; 1: 1–231.

Perth Cardiovascular Health Study: Anderson CS, Jamrozik KD, Broadhurst RJ, Stewart-Wynne EG. Predicting survival for 1 year among different subtypes of stroke: results from the Perth Community Stroke Study. *Stroke* 1994; 25: 1935–44.

Puerto Rico Health Heart Program: Garcia-Palmieri MR, Costas R Jr. Risk factors of coronary heart disease: a prospective epidemiologic study in Puerto Rico. *Prog Cardiol* 1986; Chapter 6: 101–90.

Tecumseh Community Health Study: Higgins M, Keller JB. Cholesterol, coronary heart disease, and total mortality in middle-aged and elderly men and women in Tecumseh. *Ann Epidemiol* 1992; 2: 69–76.

US Health Professionals Follow-up Study: Ascherio A, Rimm EB, Hernan MA, et al. Intake of potassium, magnesium, calcium, and fiber and risk of stroke among US men. *Circulation* 1998; 98: 1198–204.

US Nurses' Health Study (NHS-II): Stampfer MJ, Willett WC, Colditz GA, Speizer FE, Hennekens CH. A prospective study of past use of oral contraceptive agents and risk of cardiovascular diseases. *N Engl J Med*. 1988; 319(20): 1313–7.

ASIA: 26 studies

Aito Town Study: Maegawa H, Hidaka H, Okayama A, et al. Risk factors for non-insulin dependent diabetes mellitus in a rural Japanese population–incidence and risk factors. *J Japan Diab Soc*. 1992; 35: 241–8. [in Japanese with English abstract]

Akabane Study: Okada H, Horibe H, Yoshiyuki O, et al. A prospective study of cerebrovascular disease in Japanese rural communities, Akabane and Asahi. Part 1: evaluation of risk factors in the occurrence of cerebral hemorrhage and thrombosis. *Stroke* 1976 Nov-Dec; 7(6): 599–607.

Anzhen 2 Study: *J of Cardiovasc and Pulmon Disease* 1992; 11: 132–6. [in Chinese]

Beijing Aging: Tian X, Tang Z, Jiang J, et al. Effects of smoking and smoking cessation on life expectancy in an elderly population in Beijing, China, 1992-2000: an 8-year follow-up study. *J Epidemiol.* 2011; 21(5): 376–84.

Civil Service Workers: Tamakoshi A, Ohno Y,Aoki K, et al. Depressive mood and suicide among middle-aged workers: findings from a prospective cohort study in Nagoya, Japan*. J Epidemiol.* 2000; 10: 173–8.

Electricity Generating Authority of Thailand (EGAT): Vathesatogkit P, Woodward M, Tanomsup S, et al. Cohort Profile: The electricity generating authority of Thailand study, *Int J Epidemiol* 2012; 41:–359-65.

Fangshan Cohorts: Gu DF, Wu X, Li J. The Beijing Fangshan cardiovascular prevention program. *Chin J. Cardiol.* 1998; 26(2): 105–7. [In Chinese with English abstract].

Guangzhou Occupational Cohort: Lam TH, Jiang CQ, Zhang WS, et al. Smoking, occupational exposure and mortality in workers in Guangzhou, China. *Annals of Epidemiology* 2002; 12: 370–7.

Hong Kong Study: Ho SC, Woo J, Sham A' Donnan SPB. Blood pressure and 40-month mortality in elderly Chinese subjects aged 70 years and over. *J Human Hypertens.*1992; 6: 299–304.

Ikawa Study: Shimamoto T, Komachi Y, Inada H, et al. Trends for coronary heart disease and stroke and their risk factors in Japan. *Circulation* 1989; 79: 503–15.

Japan Railways: Tomita M, Yamaguchi M, Sakuma K, Gitsukawa H, Saito N, Nabutomo K. Risk factors of ischemic heart disease in middle-aged Japanese males. *J Transportation Med* 1987; 41: 30–3. [in Japanese]

Korean Medical Insurance Corporation (KMIC): Suh I, Jee SH, Kim HC, et al. Low serum cholesterol and haemorrhagic stroke in men: Korea Medical Insurance Corporation Study. *Lancet* 2001; 357(9260): 922–5.

Kounan Study: Choudhury SR, Okayama A, Kita Y, et al. The associations between alcohol drinking and dietary habits and blood pressure in Japanese men. *J Hypertens.* 1995; 13: 587–93.

Kyowa Studies: Yokota K, Harada M, Wakabayashi Y, et al. Evaluation of a community-based health education program for salt reduction through media campaigns. Nihon Koshu Eisei Zasshi. 2006 Aug;53(8):543-53. [In Japanese].

Miyama Study: Morioka S. A cohort study on the relationship between lifestyles and mortality. *Jap J of Pub Health*. [Nippon Koshu Eisei Zasshi] 1996; 43(6): 469–478.

Noichi Studies: Iso H, Naito Y, Sato S, et al. Serum triglycerides and risk of coronary heart disease. *Am J Epidemiol* 2001; 153: 490–9.

Ohasama Study: Tsuji I, Imai Y, Nagai K, et al. Proposal of reference values for home blood pressure measurement: prognostic criteria based on a prospective observation of the general population in Ohasama, Japan. *Am J Hypertens* 1997; 10: 409–18.

Saitama Cohort Study: Imai K, Matsuyama S, Miyake S, Suga K, Nakachi K. Natural cytotoxic activity of peripheral-blood lymphocytes and cancer incidence: an 11-year follow-up study of a general population. *Lancet* 2000; 356: 1795–9.

Shibata Study: Nakayama T, Date C, Yokoyama T, Yoshiike N, Yamaguchi M, Tanaka HA. 15·5-year follow-up study of stroke in a Japanese provincial city: the Shibata Study. *Stroke* 1997; 28: 45–52.

Shigaraki Town Study: Choudhury SR, Yoshida Y, Kita Y, et al. Association between electrocardiographic ischemic abnormalities and ischaemic heart disease risk factors in a Japanese population. *J Hum Hypertens.* 1996; 10: 225–34.

Shirakawa Study: Horibe H. *Nichijunkyou-shi* 1992; 26:191–7. [In Japanese]

Singapore Heart & Thyroid Study: Lee J, Heng D, Chia KS, et al. Risk factors and incident coronary heart disease in 39 Chinese, Malay and Asian Indian males: The Singapore Cardiovascular Cohort Study. *Int J Epidemiol*. 2001; 30: 983–8.

Singapore National Health Survey 1992 (NHS92): Tan CE, Emmanuel SC, Tan BY, Jacob E. Prevalence of diabetes and ethnic differences in cardiovascular risk factors. The 1992 Singapore National Health Survey. *Diabetes Care.*1999; 22(2): 241–7.

Tanno and Soubetsu Studies: Takagi S, Saitoh S, Nakano M, et al. Relationship between blood pressure level and mortality rate: an 18-year study conducted in two rural communities in Japan. *J Hypertens*. 2000; 18(2): 139–44.

Two Township Study in Taiwan (CVD FACTS): Yeh CJ, Pan WH, Jong YS, et al. (2001) Incidence and predictors of isolated systolic hypertension and isolated diastolic hypertension in Taiwan. *J Formos Med Assoc.* 2001; 100(10): 668–75.

Yunnan Tin Miner Study: Qiao YL, Taylor PR, Yao SX, et al. Relation of radon exposure and tobacco use to lung cancer among tin miners in Yunnan Province, China. *Am J Ind Med.* 1989; 16(5): 511–21.

Webtable 2: Baseline chatacteristics of men and women with and without diabetes at study recruitment

	Men		Women	
	Diabetes	No diabetes	Diabetes	No diabetes
No. participants	28 450	540 075	14 001	398 267
Mean (SD)				
Age, years	50.2 (9.8)	45.5 (9.8)	51.0 (11.1)	45.5 (8.4)
SBP, mmHg	134.6 (17.4)	132.5 (17.3)	132.3 (18.1)	127.5 (14.9)
DBP, mmHg	83.9 (11.2)	82.2 (11.1)	79.7 (11.0)	78.0 (9.6)
BMI, kg/m²	25.0 (3.2)	24.6 (3.2)	26.0 (4.1)	24.1 (2.8)
Total cholesterol, mmol/L	5.5 (1.2)	5.6 (1.2)	5.5 (1.2)	5.5 (1.0)
Smoking habit, %				
Current cigarette	27.0	35.5	14.7	18.9
Other*	50.1	40.3	24.7	22.9
Never	22.9	24.2	60.7	58.2
No. participants with measured HDL cholesterol	3767	92 870	2832	68 872
Total cholesterol, mmol/L	5.5 (1.2)	5.7 (1.2)	5.6 (1.2)	5.7 (1.0)
HDL cholesterol, mmol/L	1.2 (0.4)	1.2 (0.4)	1.3 (0.4)	1.5 (0.3)
Total cholesterol/ HDL ratio	5.3 (1.9)	4.9 (1.9)	4.6 (1.4)	4 (1.6)

Estimates are standardised for baseline age and study

* "Other“ includes ex-smoker of any type of tobacco, smoking status not known (not recorded or not able to reliably distinguish between never, ex or current), or current smoker of other types of tobacco (n=23 475; 7.2%)

Web table 3: Relevance of: (a) total cholesterol; (b) SBP; and (c) BMI to occlusive vascular mortality at ages 35-89 years, by sex and diabetes at study recruitment

		RR (95% CI)
**Cholesterol (per mmol/L)**		
Men	Diabetes	1.56 (1.37-1.76)
	No diabetes	1.56 (1.44-1.69)
Women	Diabetes	1.36 (1.19-1.56)
	No diabetes	1.24 (1.10-1.39)
**SBP (per 20 mmHg)**		
Men	Diabetes	1.54 (1.38-1.71)
	No diabetes	1.82 (1.68-1.97)
Women	Diabetes	1.45 (1.12-1.88)
	No diabetes	1.92 (1.81-2.03)
**BMI (per 5 kg/m^2)**		
Men	Diabetes	1.35 (1.26-1.44)
	No diabetes	1.38 (1.28-1.48)
Women	Diabetes	1.24 (1.15-1.34)
	No diabetes	1.21 (1.13-1.31)

RR=death rate ratio; CI=confidence interval. SBP=Systolic blood pressure; BMI=Body-mass index. Analyses are adjusted for age at risk, study, smoking and, where appropriate, total cholesterol, SBP, DBP and BMI. Usual total cholesterol and SBP refer to the long-term average level of that risk factor. Regression dilution ratios of 0·65 for total cholesterol and 0·67 for SBP were calculated by regressing serial measurements from 175,000 participants with at least one re-measurement a few years later, on baseline levels of these risk factors. No adjustment was applied for BMI, since one single measurement at baseline was highly correlated with long-term BMI. Analyses exclude those with prior ischaemic heart disease or stroke diagnosed by a doctor, and those with missing covariate data. Occlusive vascular mortality includes death from ischaemic heart disease (ICD-9 410-414), ischaemic stroke (ICD-9 433-434) or other atherosclerotic diseases (ICD-9 440, 443, 445) as the underlying cause.

Webfigure 1: Sex−specific prevalence of diabetes at study recruitment by baseline characteristics

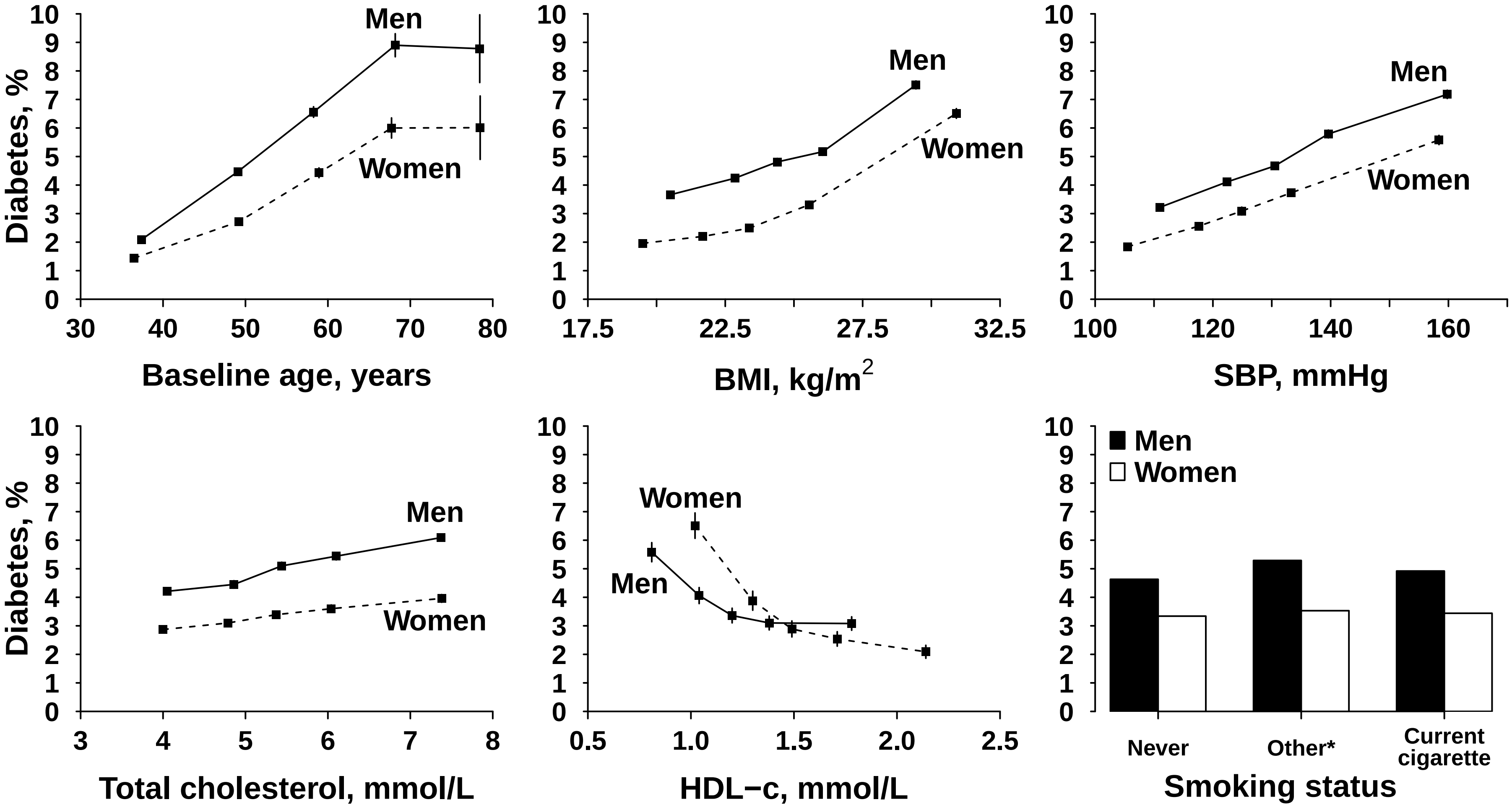

BMI=body−mass index; SBP=systolic blood pressure; HDL−c=high density lipoprotein cholesterol. *includes former smokers, smokers of non−cigarette tobacco, and people with smoking status not known.
Analyses adjusted for baseline age, cohort and smoking (where appropriate). Associations of diabetes with total cholesterol and SBP are additionally adjusted for BMI.
For the subset of 168,341 participants with HDL−c available, the overall prevalence of diabetes at recruitment was 4.1% in both men and women.

Webfigure 2: Age and sex−specific relevance of diabetes at study recruitment to IHD and ischaemic stroke mortality

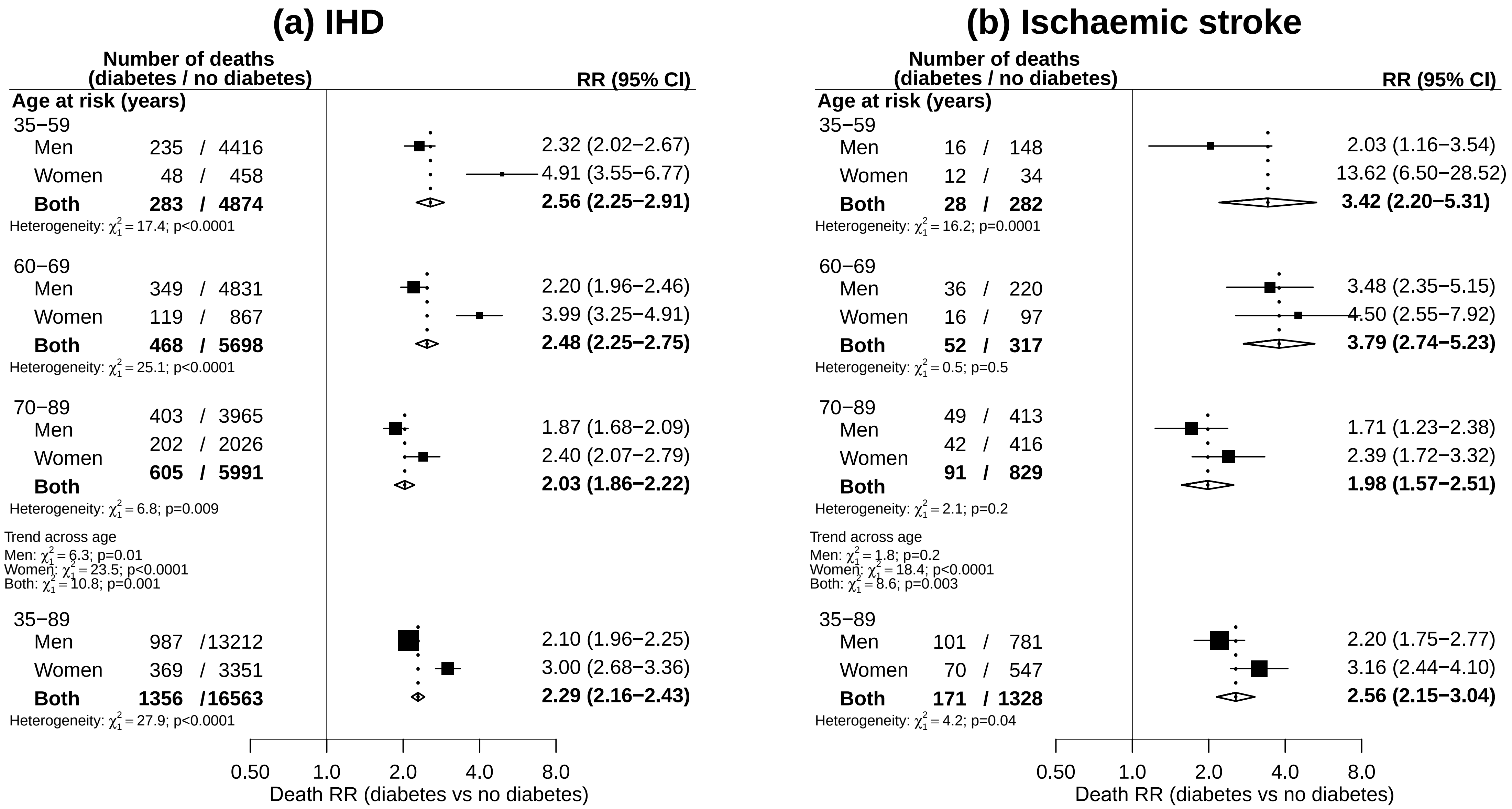

RR=rate ratio; CI=Confidence Interval. Analyses are adjusted for age at risk, BMI, SBP, DBP, total cholesterol, smoking ("never" smoked any type of tobacco regularly, "current cigarette" smoker, and "former and other", including ex−smoker of any type of tobacco, current smoker of other types of tobacco, or smoking status not known) and study, and exclude people with prior ischaemic heart disease or stroke diagnosed by a doctor, or with missing covariate data. Each diamond represents the inverse variance−weighted average of the two estimates above it. Ischaemic heart disease includes (ICD−9 410−414) and ischaemic stroke includes (ICD−9 433−434).

Webfigure 3: Age and sex−specific relevance of diabetes at study recruitment to occlusive vascular mortality (limited to studies that included both men and women)

Age at risk (years)	Number of deaths (diabetes / no diabetes)	RR (95% CI)
**35−59**		
Men	174 / 3007	2.19 (1.87−2.58)
Women	52 / 478	5.18 (3.79−7.06)
**Both**	**226 / 3485**	**2.54 (2.20−2.92)**
Heterogeneity: $\chi^2_1 = 22.9$; p<0.0001		
**60−69**		
Men	227 / 2779	2.29 (1.99−2.64)
Women	121 / 911	4.00 (3.26−4.91)
**Both**	**348 / 3690**	**2.69 (2.39−3.02)**
Heterogeneity: $\chi^2_1 = 19.5$; p<0.0001		
**70−89**		
Men	235 / 3118	1.68 (1.46−1.92)
Women	246 / 2479	2.35 (2.05−2.69)
**Both**	**481 / 5597**	**1.97 (1.78−2.16)**
Heterogeneity: $\chi^2_1 = 11.7$; p=0.0006		
Trend across age: Men: $\chi^2_1 = 7.1$; p=0.008; Women: $\chi^2_1 = 30.8$; p<0.0001; Both: $\chi^2_1 = 12.5$; p=0.0004		
**35−89**		
Men	636 / 8904	2.02 (1.86−2.19)
Women	419 / 3868	2.89 (2.60−3.21)
**Both**	**1055 / 12772**	**2.29 (2.14−2.44)**
Heterogeneity: $\chi^2_1 = 27.1$; p<0.0001		

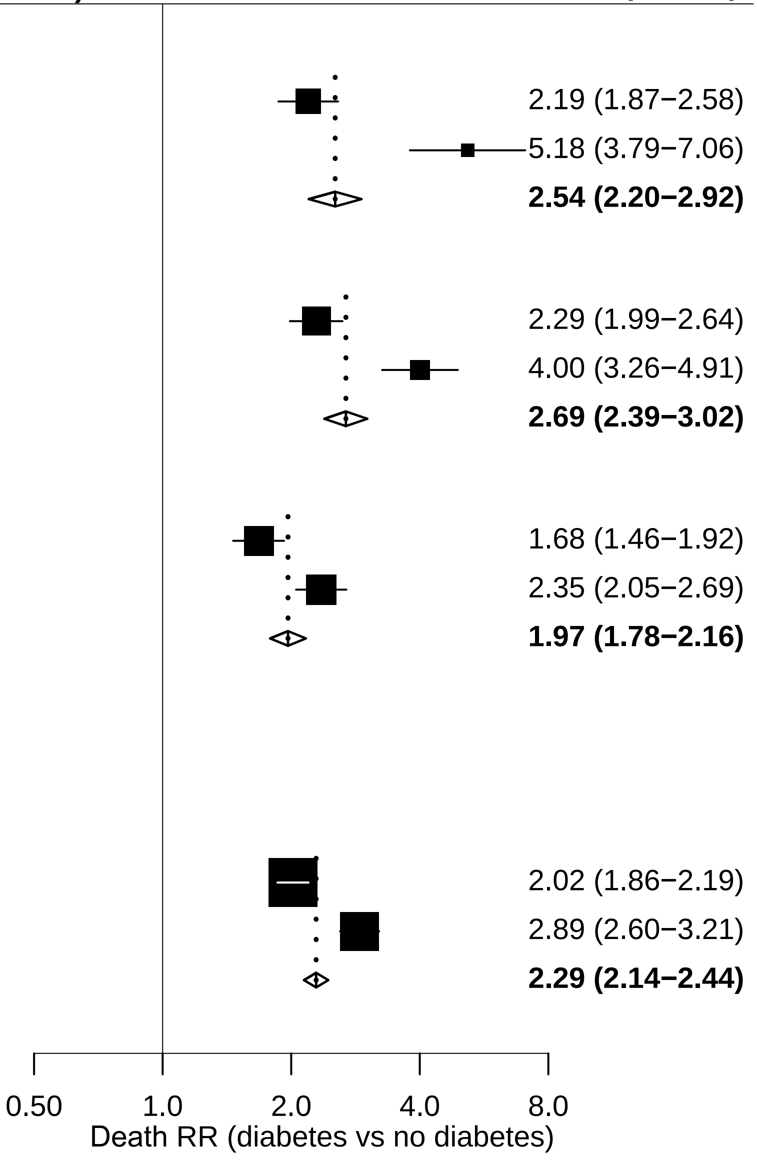

RR=rate ratio; CI=confidence interval. Analyses are adjusted for age at risk, BMI, SBP, DBP, total cholesterol, smoking ("never" smoked any type of tobacco regularly, "current cigarette" smoker, and "former and other", including ex−smoker of any type of tobacco, current smoker of other types of tobacco, or smoking status not known), and study, and exclude people with prior ischaemic heart disease or stroke diagnosed by a doctor, or with missing covariate data. Each diamond represents the inverse variance−weighted average of the two estimates above it. Occlusive vascular mortality includes death from ischaemic heart disease (ICD−9 410−414), ischaemic stroke (ICD−9 433−434) or other atherosclerotic diseases (ICD−9 440, 443, 445) as the underlying cause.

Webfigure 4: Relevance of diabetes at study recruitment to occlusive vascular mortality, by age and sex in those with prior IHD or stroke

Age at risk (years)	Number of deaths (diabetes / no diabetes)	RR (95% CI)
**35−59**		
Men	82 / 757	1.83 (1.44−2.34)
Women	13 / 93	2.91 (1.52−5.57)
**Both**	**95 / 850**	**1.92 (1.53−2.40)**
Heterogeneity: $\chi^2_1 = 1.7$; p=0.2		
**60−69**		
Men	166 / 1236	1.81 (1.51−2.16)
Women	66 / 270	3.53 (2.60−4.80)
**Both**	**232 / 1506**	**2.10 (1.81−2.45)**
Heterogeneity: $\chi^2_1 = 13.9$; p=0.0002		
**70−89**		
Men	175 / 1171	1.58 (1.33−1.87)
Women	106 / 667	1.81 (1.46−2.24)
**Both**	**281 / 1838**	**1.66 (1.45−1.90)**
Heterogeneity: $\chi^2_1 = 0.9$; p=0.3		
**35−89**		
Men	423 / 3164	1.72 (1.55−1.92)
Women	185 / 1030	2.25 (1.90−2.66)
**Both**	**608 / 4194**	**1.85 (1.69−2.03)**
Heterogeneity: $\chi^2_1 = 6.9$; p=0.009		

Trend across age groups:
Men: $\chi^2_1 = 1.3$; p=0.3
Women: $\chi^2_1 = 9.5$; p=0.002
Both: $\chi^2_1 = 2.7$; p=0.10

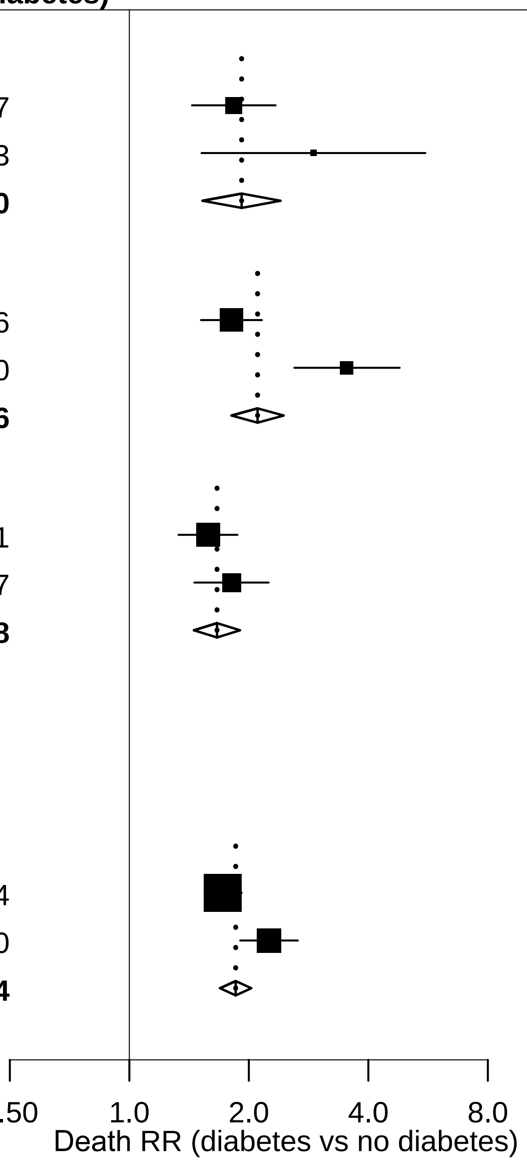

RR=rate ratio; CI=confidence interval. Analyses are adjusted for age at risk, BMI, SBP, DBP, total cholesterol, smoking ("never" smoked any type of tobacco regularly, "current cigarette", smoker; and "former and other", including ex−smoker of any type of tobacco, current smoker of other types of tobacco, or smoking status not known) and study, and exclude people without prior ischaemic heart disease or stroke diagnosed by a doctor, or with missing covariate data. Each diamond represents the inverse variance−weighted average of the two estimates above it. Occlusive vascular mortality includes death from ischaemic heart disease (ICD−9 410−414), ischaemic stroke (ICD−9 433−434) or other atherosclerotic diseases (ICD−9 440, 443, 445) as the underlying cause.

Webfigure 5: Relevance of diabetes at study recruitment to occlusive vascular mortality at ages 35−89, by sex and region

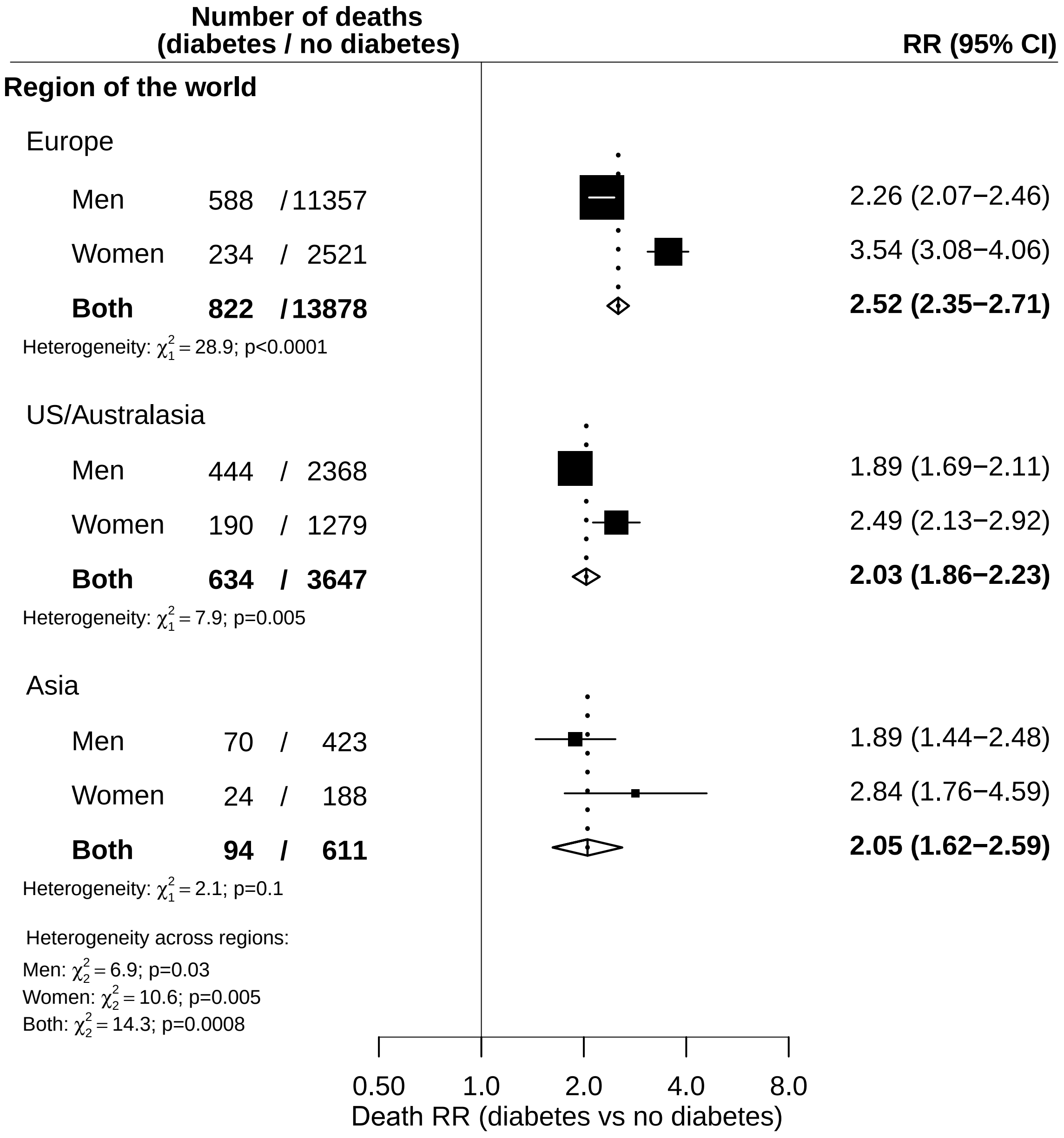

RR=rate ratio; CI=confidence interval. Analyses are adjusted for age at risk, BMI, SBP, DBP, total cholesterol, smoking (“never” smoked any type of tobacco regularly, “current cigarette”, smoker; and “former and other”, including ex−smoker of any type of tobacco, current smoker of other types of tobacco, or smoking status not known) and study, and exclude people with prior ischaemic heart disease or stroke diagnosed by a doctor, or with missing covariate data. Each diamond represents the inverse variance−weighted average of the two estimates above it. Occlusive vascular mortality includes death from ischaemic heart disease (ICD−9 410−414), ischaemic stroke (ICD−9 433−434) or other atherosclerotic diseases (ICD−9 440, 443, 445) as the underlying cause.
